# Freestyle-Like V-Y Flaps of the Eyebrow: A New Outlook and Indication of an Historical Technique

**DOI:** 10.1155/2013/182518

**Published:** 2013-10-21

**Authors:** Angelo Alberto Leto Barone, Matteo Rossi, Gabriele Giunta, Marco Carmisciano, Luigi Di Rosa, Francesca Toia, Salvatore D'Arpa, Adriana Cordova

**Affiliations:** Department of Surgical, Oncological and Stomatological Sciences, University of Palermo, Plastic and Reconstructive Surgery, Via del Vespro 129, 90127 Palermo, Italy

## Abstract

The eyebrow region is of utmost importance for facial movement, symmetry, and the overall cosmetic appearance of the face. Trauma or tumor resection often leave scars that may dislocate the eyebrow producing an alteration both in static symmetry of the face and in the dynamic expressivity. The authors present a technique for eyebrow's defects repair using the remaining eyebrow advancement by means of a “freestyle-like” V-Y flap. In the past two years a total of eight consecutive patients underwent excision of skin lesions in the superciliary region and immediate reconstruction with this technique. On histology, six patients were affected from basal cell carcinomas, one from squamous cell carcinoma, and one from congenital intradermal melanocytic nevus. The pedicle of the flap included perforators from the supratrochlear, supraorbital, or superficial temporalis artery. Advancement of the entire aesthetic subunit that includes the eyebrow using a V-Y perforator flap was performed successfully in all cases achieving full, tension-free closure of defects up to 3.0 cm. “Freestyle-like” V-Y flaps should be considered as a first-line choice for partial defects of the eyebrow. The greater mobility compared to random subcutaneous flaps allows to reconstruct large defects providing an excellent cosmetic result.

## 1. Introduction

Lesions of the superciliary arch are common during facial trauma, as the sharp bony prominences can easily lacerate the overlying skin. In contrast, tumoral lesions are quite rare and represent, according to our experience, about 6% of the tumors involving the periorbital region. Due to the relative rarity of such tumors in the superciliary arch, very little has been published regarding this topic, and there are no guidelines for reconstruction. The majority of the reconstructive techniques of the eyebrow are reported as case reports and not as case series. 

In this paper, we review the most recent literature on eyebrow reconstruction and we present the authors' technique for eyebrow repair using the remaining eyebrow advancement by means of a “freestyle-like” V-Y flap. This technique allows the repair of extensive defects (up to 50% of the entire eyebrow) with excellent cosmetic outcomes. 

The advantages of using V-Y flaps have been widely described for the reconstruction of small to medium defects of the face following postoncologic resection of skin tumors. The senior authors have already published in the 1980s and 1990s their first experiences on the use of flaps with a subcutaneous pedicle in the upper and lower eyelid regions [1–3].

In 2009, Marchac et al. have published an article on lower eyelid reconstruction with a subcutaneous pedicled flap highlighting the important role of this technique [4], which was already in use but which had disappeared from the most recent publications. As of today, the introduction and widespread use of mobilization techniques of freestyle flaps on perforator vessels has allowed us to review the mobilization technique of the V-Y flaps and reconsider the use of such flaps, renovating the interest toward this technique. 

 The most recent surgical applications of this technique [5–7] have shown that a thick pedicle, including the entire subcutaneous tissue, is no longer necessary to advance a V-Y flap. Rather, it is currently possible to mobilize wide portions of tissue that are pedicled just on perforators. 

These free stile V-Y flaps are safer, with a thinner pedicle and with a greater advancement potential for reconstruction of different body areas, including the superciliar region. 

## 2. Materials and Methods

### 2.1. Patient Population

In the last two years eight patients, six males and two females (mean age: 64.25; median age: 69; range: 35–82), underwent excision of skin lesions in the superciliary region. On histology, five of these patients were affected from nodular basal cell carcinomas, one from pigmented basal cell carcinomas, one from residual squamous cell carcinoma following prior excision, and one from congenital intradermal melanocytic nevus (Table 1). In the latter patient, the lesion was removed for purely cosmetic reasons. In the remaining cases, surgery was performed either for treatment or for proper diagnosis. All patients underwent immediate reconstruction using a V-Y advancement flap according to the technique described below. An independent, blinded panel of three plastic surgeons was asked to evaluate the results based on photographs. Result were rated as “excellent,” “good,” “acceptable” or “bad.” 

### 2.2. Surgical Technique

The defect to be determined in the superciliary region is marked and included in a square or oval area. Excision of the tumor is obtained to establish oncologic radicality. A triangular flap including the entire hairy portion of the remaining eyebrow is then marked. The base of the flap is represented by the lateral margin of the defect, and on occasion, depending on the vertical dimension of the defect, it may also include a part of the non-hair bearing skin above (frontal region) or below the eyebrow (eyelid region).

The height of the triangle should be such to allow the closure of the donor site in a tension-free V-Y fashion. 

The flap is incised peripherally, and a careful exploration of the subcutaneous tissue begins, to identify and preserve a perforator vessel of adequate caliber for the nourishment of the cutaneous island in a freestyle fashion. 

The dissection begins from the inferior margin, where it is easier to find the branches of the supratrochlear and supraorbital arteries. The supraorbital artery, arising from the ophthalmic artery, passes through the supraorbital foramen. It divides then into a superficial branch and a deep branch, which supply the eyebrow and the forehead. If it is not affected during tumor excision, the supratrochlear artery, which lies more medial than the supraorbital vessels [8], should be preserved not to interfere with neighboring anatomic territories that may be utilized for alternative salvage flaps. 

When vessels of adequate caliber are isolated, they are preserved without the need for complete skeletonization. The subcutaneous tissue around the vessels can be removed to obtain a thinner pedicle and greater mobility but still ensuring good vascularization. Once the pedicle has been carved, the flap can advance without any tension to repair up to 50% of the entire length of the eyebrow. 

**Figure 1:**
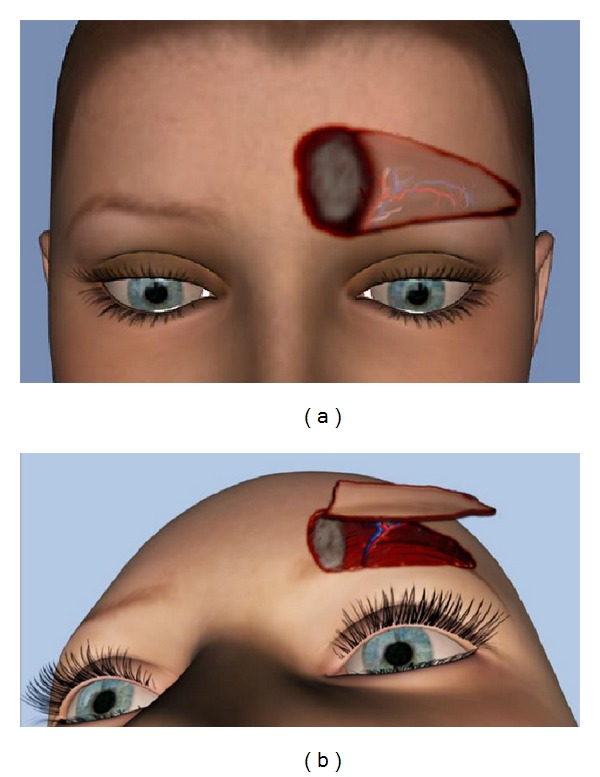
3D illustration of surgical technique: the defect is outlined in an oval area, and a triangular flap including the entire hairy portion of the remaining eyebrow is incised (a); the flap is advanced toward the difect pedicled on a perforator vessel (b).

The flap is then sutured in double layer paying attention to the correct positioning of the hair-bearing area which has to be anchored symmetrically to the contralateral brow. In some cases, quilting sutures to the periosteum can be useful to ensure the persistence of symmetry between the two orbital arches (Figure 1). 

## 3. Results

**Figure 2:**
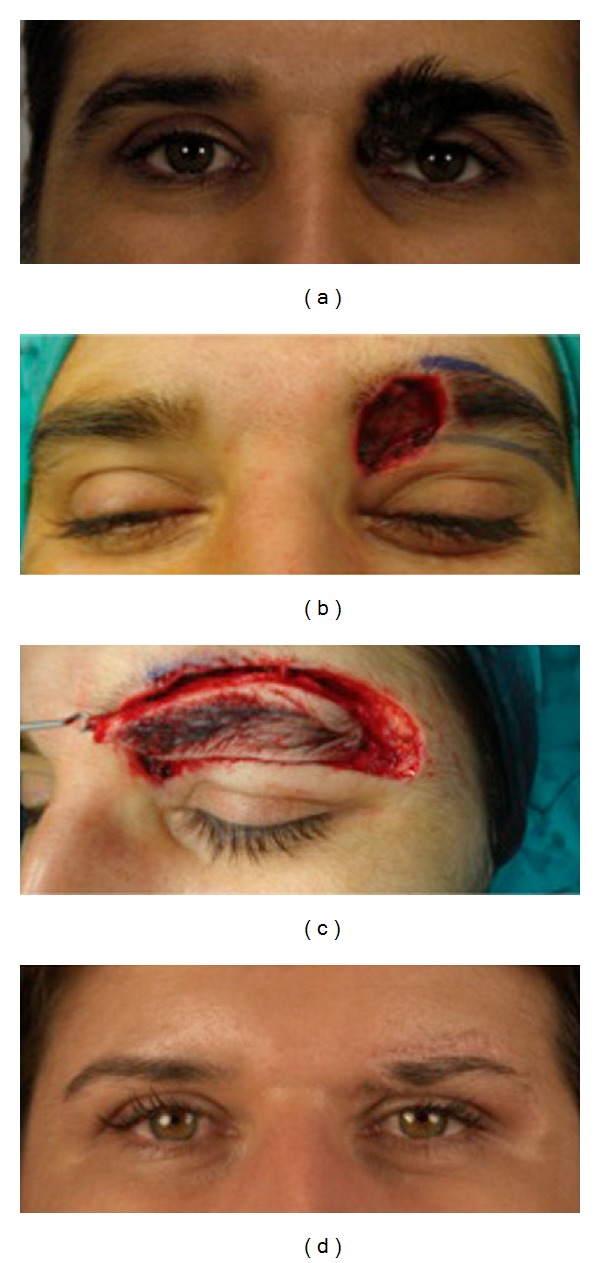
a 35-year-old female patient affected from congenital intradermal melanocytic nevus of the medial portion of the left eyebrow (a). Excision of the nevus was performed, and immediate reconstruction using a freestyle-like V-Y advancement flap was used to cover the defect (b, c). Cosmetic appearance was considered satisfactory at follow up 1-year postoperatively (d).

**Figure 3:**
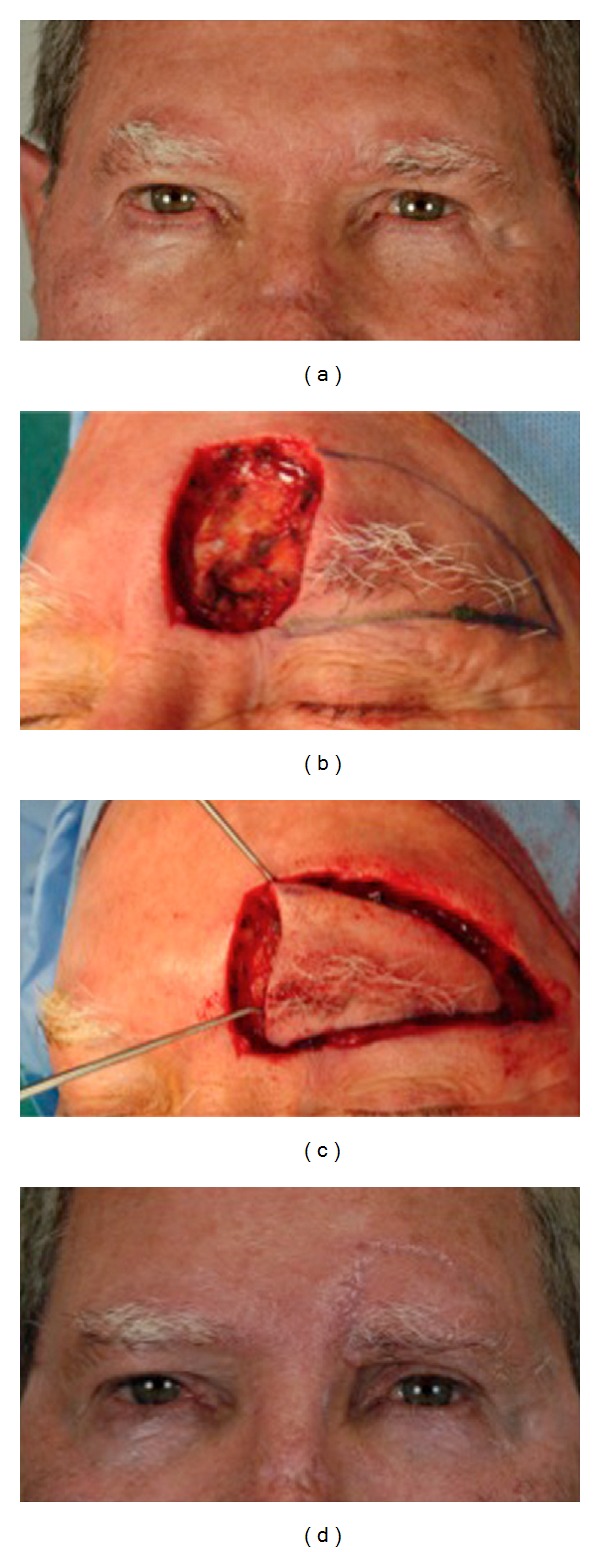
a 78-year-old male underwent squamous cell carcinoma tumor removal from the medial region of the left eyebrow. Histology showed tumor infiltrations in the margins so wide excision was planned (a). A 3 cm wide defect was produced, and a freestyle-like V-Y advancement flap was used to close the defect (b, c). Cosmetic appearance was considered satisfactory at follow up 3-year postoperatively (d).

Advancement of the entire aesthetic subunit that includes the eyebrow using a V-Y flap was performed successfully in a case series of 8 consecutive patients of partial eyebrow reconstruction, achieving full, tension-free closure of defects up to 3.0 cm in length (Figures 2 and 3). Oncological radicality was obtained in all cases. All patients were satisfied with the operation. Results of the evaluation by the panel of indipendent surgeons were “excellent” in 7 cases and “good” in 1 case. No patients had complications during a mean follow-up period of 13.5 months (Table 1). 

## 4. Discussion

Despite the aesthetic and functional relevance that the eyebrow maintains in the eumorphism of the face, the scientific literature revision on eyebrow reconstruction has highlighted a lack of efficacious and reproducible surgical techniques. 

Several techniques have been reported as case reports and not as case series, many more refer to composite grafts and flaps harvested from the scalp, and others are extremely complex and are not justified for the use because they do not provide any advantage for the patient. 

Composite grafts for eyebrow reconstruction have been reported as strip grafts [9, 10], punch grafts [10, 11], and hair micrografts from the scalp or the nuchal area [12, 13]. 

Punch and micrografts cannot be implated immediately during the removal of a tumor and they are indicated for a scarring alopecia repair as a secondary reconstructive procedure. 

Strip grafts can allow a single-stage reconstruction of large defects but they need to be thin and narrow to receive adequate vascularization from the edges and the wound bed. 

 Flaps from the scalp that are pedicled on parietal branches of the superficial temporal vessels have been described for defects involving part of the entire eyebrow. Elevation of such flaps is tedious as dissection and tunneling of a long pedicle are required. 

The authors believe that strip grafts or local flaps from the scalp represent always an aesthetic compromise and should be reserved to defects involving the entire eyebrow.

Kim et al. have recently published a case report on an elegant microsurgical reconstruction with a supraorbital artery perforator free flap sculpted from the contralateral eyebrow and anastomosed in an end to end fashion to the supraorbital vessels of the recipient site for the repair of a 1.7 cm long posttraumatic defect [14]. We certainly agree with the concept that a technique should not be chosen just because it is easier, but we believe that we need to take in high consideration the quality of the result. However, when comparable results can be achieved, techniques that are easier to perform should be preferred, especially in partial postoncological reconstructions and in elderly patients in which complex microsurgical reconstructions are not indicated [15]. 

Among the most common techniques for partial eyebrow defects reconstruction, the use of a double-Z rhombic technique has been reported [16]. This technique, however, is responsible for larger scars in the hair-bearing area as well as in the glabrous skin above and below the eyebrow. 

More recent and innovative is a case report in which the authors use a perforator-like flap based on the supratrochlear artery advanced in a medial to lateral direction for partial reconstruction of the lateral half of the eyebrow [17]. In this case, the cosmetic result is excellent, and we agree to the principle of mobilizing the entire aesthetic subunit in a single advancement flap. We came to the conclusion, however, that for the mobilization of relatively small areas, such as the eyebrow, it is not necessary to perform a fine skeletonization of the perforator. Moreover, in contrast with the experience of some authors [18], we were able to close defects larger than 1.7 cm up to 3 cm using our method.

The reconstructed eyebrow, despite being symmetric and well-oriented, will necessarily be shorter than the contralateral brow. As understandable, this is due to the fact that no additional hair-bearing areas are transferred. However, very often the reduction of 30–50% in length of the brow, especially in elderly patients, does not result in a noticeable cosmetic defect. 

This flap advances better in the lateral-to-medial direction, as the lack of hair in the lateral third of the brow is cosmetically less noticeable. For this reason, this technique is our elective method to repair defects of the medial third of the eyebrow. Nevertheless, this technique could be easily utilized for lateral defects reconstruction. In such cases, however, the distance between the two glabellar areas will increase. 

 The common thinking that is now widespread following the introduction of free-style perforator flaps has determined a new approach on the mobilization of advancement V-Y flaps allowing to take distance from the historical concept of random-vascularization flaps. 

In the case of V-Y flaps of the eyebrow here described, the greater mobility compared to random subcutaneous flaps allows to obtain easily the correct orientation of the hair-bearing areas. 

The scars resulting from a V-Y flap do not involve hair-bearing zones and are parallel to the minimum tension lines of the forehead and the eyelid, resulting in nonvisible scars with scar maturation. 

## 5. Conclusions

“Freestyle-like” V-Y flaps should be considered potentially as a first-line choice for partial defects of the eyebrow. Undoubtedly, this technique reduces the length of the eyebrow and is indicated for defects involving between 30 and 50% providing an excellent cosmetic result.

Grafts and flaps harvested from the scalp, due to differences in annexa presence, hair orientation and growth length, should be reserved to total and subtotal reconstructions. 

## Figures and Tables

**Table 1:** Series of patients treated with a freestyle-like V-Y advancement flap for eyebrow reconstruction.

Number	Sex	Age	Diagnosis	Defect size *H* (cm) × *W* (cm)	Complications
1	M	76	Nodular basal cell carcinoma	1.8 × 2.2	None
2	M	82	Nodular basal cell carcinoma	1.9 × 2.8	None
3	M	74	Pigmented basal cell carcinoma	2.2 × 3	None
4	F	35	Congenital intradermal melanocytic nevus	1.9 × 2.7	None
5	M	60	Nodular basal cell carcinoma	2.1 × 1.9	None
6	F	45	Nodular basal cell carcinoma	2.1 × 2.6	None
7	M	78	Squamous cell carcinoma	3 × 2.4	None
8	M	64	Nodular basal cell carcinoma	1.7 × 2.3	None
